# Markov chains as a proxy for the predictive memory representations underlying mismatch negativity

**DOI:** 10.3389/fnhum.2023.1249413

**Published:** 2023-09-13

**Authors:** Erich Schröger, Urte Roeber, Nina Coy

**Affiliations:** ^1^Wilhelm Wundt Institute for Psychology, Leipzig University, Leipzig, Germany; ^2^Max Planck School of Cognition, Leipzig, Germany

**Keywords:** regularity representation, memory, Markov model, perception, adaptation, predictive processing, mismatch negativity

## Abstract

Events not conforming to a regularity inherent to a sequence of events elicit prediction error signals of the brain such as the Mismatch Negativity (MMN) and impair behavioral task performance. Events conforming to a regularity lead to attenuation of brain activity such as stimulus-specific adaptation (SSA) and behavioral benefits. Such findings are usually explained by theories stating that the information processing system predicts the forthcoming event of the sequence via detected sequential regularities. A mathematical model that is widely used to describe, to analyze and to generate event sequences are Markov chains: They contain a set of possible events and a set of probabilities for transitions between these events (transition matrix) that allow to predict the next event on the basis of the current event and the transition probabilities. The accuracy of such a prediction depends on the distribution of the transition probabilities. We argue that Markov chains also have useful applications when studying cognitive brain functions. The transition matrix can be regarded as a proxy for generative memory representations that the brain uses to predict the next event. We assume that detected regularities in a sequence of events correspond to (a subset of) the entries in the transition matrix. We apply this idea to the Mismatch Negativity (MMN) research and examine three types of MMN paradigms: classical oddball paradigms emphasizing sound probabilities, between-sound regularity paradigms manipulating transition probabilities between adjacent sounds, and action-sound coupling paradigms in which sounds are associated with actions and their intended effects. We show that the Markovian view on MMN yields theoretically relevant insights into the brain processes underlying MMN and stimulates experimental designs to study the brain’s processing of event sequences.

## Introduction

During the last decades the predictive nature of the brain’s information processing has been increasingly emphasized, acknowledging that the processing of a current event is—to a large extent—determined by the already established knowledge, memory or model of the world (Gregory, 1980; Mumford, 1992; Friston, 2005; Bar, 2007; Ahissar et al., 2009; Clark, 2015). Considering that it is used in a predictive manner, this memory is sometimes called “generative model.” Research studying the establishment, representation and application of such generative models typically presents sequences of events (stimuli) to participants. Event sequences are constructed in a way that they conform to one or several rules defining the succession of events, and it is tested how events are processed, depending on whether they do or do not conform to the rule(s). A consistent finding is that brain and behavioral measures reveal processing differences between rule conforming and rule violating events. They indicate facilitation in the processing of rule-conforming and a hindrance in the processing of rule-violating events. At the behavioral level, facilitation manifests as benefits such as increased accuracy in task performance, whereas the hindrance manifests as costs such as prolongation of response times (Parmentier, 2014). At the brain level, facilitation pays out in various forms of attenuation of brain activity, for example, stimulus-specific adaptation (SSA) in neural firing rates (Nelken, 2014; Carbajal and Malmierca, 2018; Willmore and King, 2023), N1-suppression (Horváth, 2015; Schröger et al., 2015; Auksztulewicz and Friston, 2016) or repetition positivity (Haenschel et al., 2005; Costa-Faidella et al., 2011) in the event-related potential (ERP), whereas hindrance is indicated by various prediction error signals such as the Mismatch Negativity (MMN) or the P3a (Näätänen, 1990; Bendixen et al., 2012).

**Figure 1:**
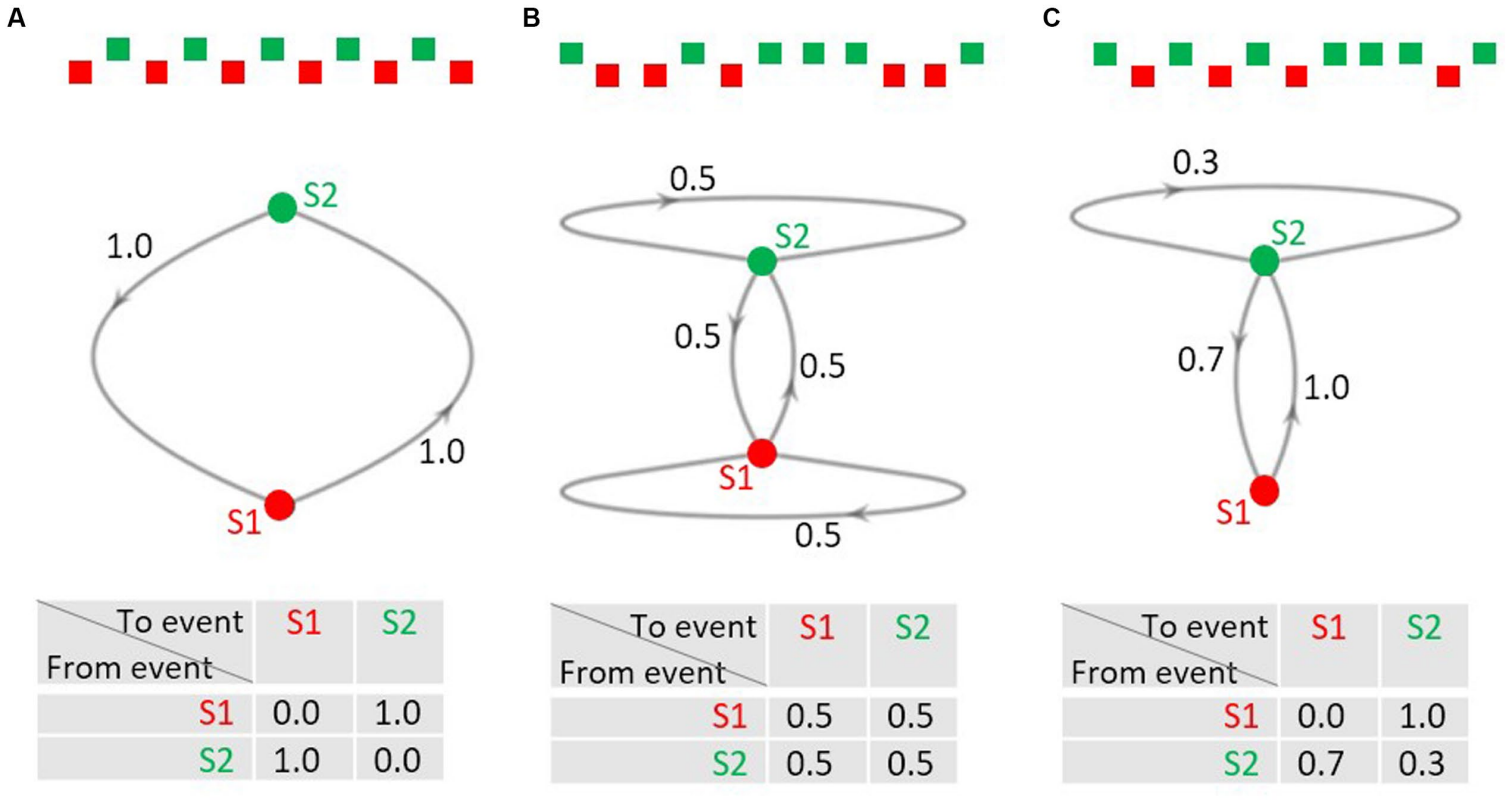
**(A–C)** Examples of 2-state Markov chains. Upper row: excerpts from event sequences; middle row: directed graphs of the Markov chains. S1 and S2 denote the states, the numbers at the arrows denote the probability of a transition from the state from which the arrow emerges to the state the arrow points to. Note that transitions with a probability of 0.0 are not shown for ease of display. Lower row: transition probability matrices. The probabilities in each row must add up to 1.0.

Sequences of events are not only relevant in research on the predictive brain but also in the concept of Markov chains, a mathematical model concerned with the description, analysis, and generation of event sequences (Markov, 1913; Hayes, 2013). In Markov jargon, the possible events in a sequence are called the states of the system. The Markov chain is the process of the trajectory of the states over time. An interesting feature of Markov chains is that only the current state of the system determines what state(s) the system can change to at the next point in time; the trajectory on how the current state emerged does not matter (Markov property); in this sense a Markov chain is memoryless. Thus, the distribution of the probabilities of the possible states at the next stage is fully described by the probabilities of the possible transitions between the current and the next state. Consider the simple case that a sequence consists of two alternating events A and B (Figure 1A): the transition probability from event A to B equals the transition probability from event B to A, both being 1.0. As A does never follow A, and B never B, the respective transition probabilities are 0.0. Markov chains can be visualized as directed graphs (middle row of Figure 1) with the points from which the arrows emerge or at which they arrive being the possible states. The transition probabilities can also be listed in a transition probability matrix that contains the transition probabilities for all possible changes from one possible state into another (bottom row of Figure 1).

The transition probability matrix in Figure 1B describes a sequence, in which any of the four possible transitions is of equal probability, that is, it is equally likely to move from A to B as from A to A, and equally likely to move from B to A as from B to B. Please note, that the row sums of the matrix must add to 1.0, whereas the column sums do not need to add to 1.0. This is shown in Figure 1C: it describes a sequence where event A is always followed by event B, whereas B can be followed by A with a probability of 0.7 and by B with a probability of 0.3. The transition probabilities for a given sequence can be computed via the counts of the actually observed transitions listed in a transition frequency matrix. Normalizing the transitions row-wise yields the transition probability matrix.

The Markov chain concept can be generalized from the first-order examples illustrated in Figure 1 to Markov chains of higher-order. For example, second-order Markov chains describe stochastic processes in which the probability of a future state is based on the two preceding states. In principle higher-order Markov chains can include an unlimited number of preceding states to predict the next state, though the more states need to be considered the more memory intensive and the less parsimonious the model becomes. In fact, higher-order Markov chains are often needed to properly model stochastic processes in domains being characterized by higher-order dependencies such as the succession of states of weather over time, the succession of words in natural language, or the development of financial markets (Hayes, 2013). The concept has also been used in (cognitive) neuroscience to model, for example, decision-making or speech recognition. It has not yet received much attention in research of predictive processes like those using MMN and research in related fields (for few exceptions see below). This paper elaborates conceptual aspects and potential benefits of such a Markovian view on the MMN, and explores how Markov chains are a simple, yet useful approach to study and explain MMN. Specifically, to consider the event sequences used in MMN research as Markov chains can facilitate a better understanding of the informational basis underlying the elicitation of MMN, make implicit theoretical assumptions explicit, enable re-interpretation of existing research findings, and stimulate the design of theoretically relevant experiments. We start our elaboration by briefly introducing the field of MMN research and our rationale for regarding the Markov model as a proxy for the neural model (memory) underlying MMN elicitation.

## Mismatch negativity

A sound violating a rule inherent to the preceding sequence of sounds (e.g., most sounds are of pitch A, the rule violating sound is of pitch B) has been shown to elicit an increased negativity in frontocentral regions of the scalp of the auditory ERP at a latency of around 150 ms in comparison to a sound that conforms to the regularity commonly labeled as MMN (Näätänen, 1990; Näätänen et al., 2011). This simple frequency repetition rule is commonly referred to as the classical oddball paradigm, in which rule-conforming sounds are called standards, whereas rule-violating sounds are called deviants. Importantly, rules can be more complex than the simple sound repetition rule. For example, tones violating the sequence rules “long tones are followed by high tones, whereas short tones are followed by low tones” also elicit MMN (Paavilainen et al., 2007; Bendixen et al., 2008). MMN is accessible with neuroscientific methods other than EEG such as MEG, fMRI, NIRS, PET, human intracranial recordings (Näätänen, 1992), or animal *in vivo* whole-cell patch-clamp recordings (Valdés-Baizabal et al., 2021). It has been described for other modalities such as vision (Kimura, 2012; Stefanics et al., 2014; Czigler and Kojouharova, 2021), and even somatosensation (Butler et al., 2011; Strömmer et al., 2014) and smell (Ochiai et al., 2021). It is generated in the respective sensory cortices, but generators in frontal brain areas may also be involved (Näätänen and Alho, 1995; Escera and Malmierca, 2014; Carbajal and Malmierca, 2018).

At the core of the prevalent MMN theory is that our brain compares the neural representation of an incoming (standard or deviant) sound with the memory trace for the standard sound. If this comparison yields a mismatch, the MMN is elicited (Näätänen, 1990). Thus, the presence of MMN is taken as evidence that the features characterizing the rule must have been encoded and that a violation of this rule has been detected by the brain. Though, many MMN results can be explained by the somewhat simpler adaptation (release-of-refractoriness) hypothesis (O’Shea, 2015). According to this hypothesis, the feature-specific neurons underlying the elicitation of the N1 component of the ERP (overlapping in time with the MMN) adapt to the features of the frequently presented standard, resulting in an attenuation of the N1. The N1 in response to a deviant is (partly) elicited by feature-specific neurons that are less adapted, resulting in a relatively larger N1. When subtracting the standard ERP from the deviant ERP, the difference in the N1s emerges as the MMN. A similar claim has been made for the P2 component of the ERP, stating that the MMN emerges due to a differential state of adaptation between the P2 elicited by standards and deviants (Haenschel et al., 2005). Namely, frequently (standard) compared to rarely (deviant) encountered stimuli elicit larger P2 amplitudes, resulting in a negative difference in a deviant-minus-standard contrast. In a rabbit against hedgehog race like manner, there are many papers attempting to dismiss the adaptation hypothesis in favor of the mismatch hypothesis, which in turn, motivated the representatives of the other theory to improve the adaptation hypothesis in respective counter-publications (Winkler et al., 1993; Jääskeläinen et al., 2004; Näätänen et al., 2005; May and Tiitinen, 2010). This race is ongoing.

Although memory-comparison based accounts of MMN already acknowledged its predictive nature (Näätänen, 1992), Winkler et al. (1996) were first to explicitly characterize the mental model of the acoustic environment underlying MMN in terms of perceptual inferences. They propose two essential functions of such a model: “inferring future events from the history of auditory input and checking the acoustic stream for anomalies” (p. 240). The MMN was suggested to be an indicator of model updating in the light of new information. Winkler et al. also proposed two essential characteristics of such a model: First, it includes detected relationships between the stimuli, and second, the inferences the model can draw from a sequence of stimuli depend on how deterministic or stochastic that sequence is. In 2007, Winkler presented the regularity-violation interpretation of MMN by explicitly stating that “the auditory oddball paradigm can also be described in terms of a regular relationship between sounds” (Winkler, 2007). The predictive memory or generative model contains the respective “predictive regularity representations” (Schröger et al., 2014; Winkler and Schröger, 2015). Although this rather Markovian view on the MMN seems to be widely accepted in the community, explicit reference to Markov chains or transition probabilities is rare in MMN research (Furl et al., 2011; Koelsch et al., 2016; Mittag et al., 2016; Tsogli et al., 2019, 2022; Korka et al., 2021).

## The Markov model as a proxy for the neural model underlying MMN

According to the regularity-violation interpretation of MMN (Winkler, 2007), the rules inherent to a sound sequence, and consequently the violations of those rules, relate to the transitions between the types of sound (i.e., *states* in Markov terminology) constituting that sequence. In the simplest case there are two sound types, namely a (frequently occurring) standard and a (rare, interspersed) deviant. In a typical MMN experiment the transition probability from a standard to a standard is high, while the transition probability from a standard to a deviant is low. Or in other words, there are at least two possible transitions from one sound to the next, one of which has a larger transitional probability relative to (at least) one other. Hence, sequences of sounds in MMN experiments have properties of a Markov chain. Apart from this similarity at the descriptive level of stimulus structure, there is also a similarity between MMN theory and the Markov model on a more conceptual level. MMN theory assumes that there exists a neural or mental model hosting the detected regularities of a sound sequence. Although there is no consensus in the community on how these models look, it is often assumed that they generate predictions about forthcoming sounds (Winkler and Czigler, 2012).

**Figure 2:**
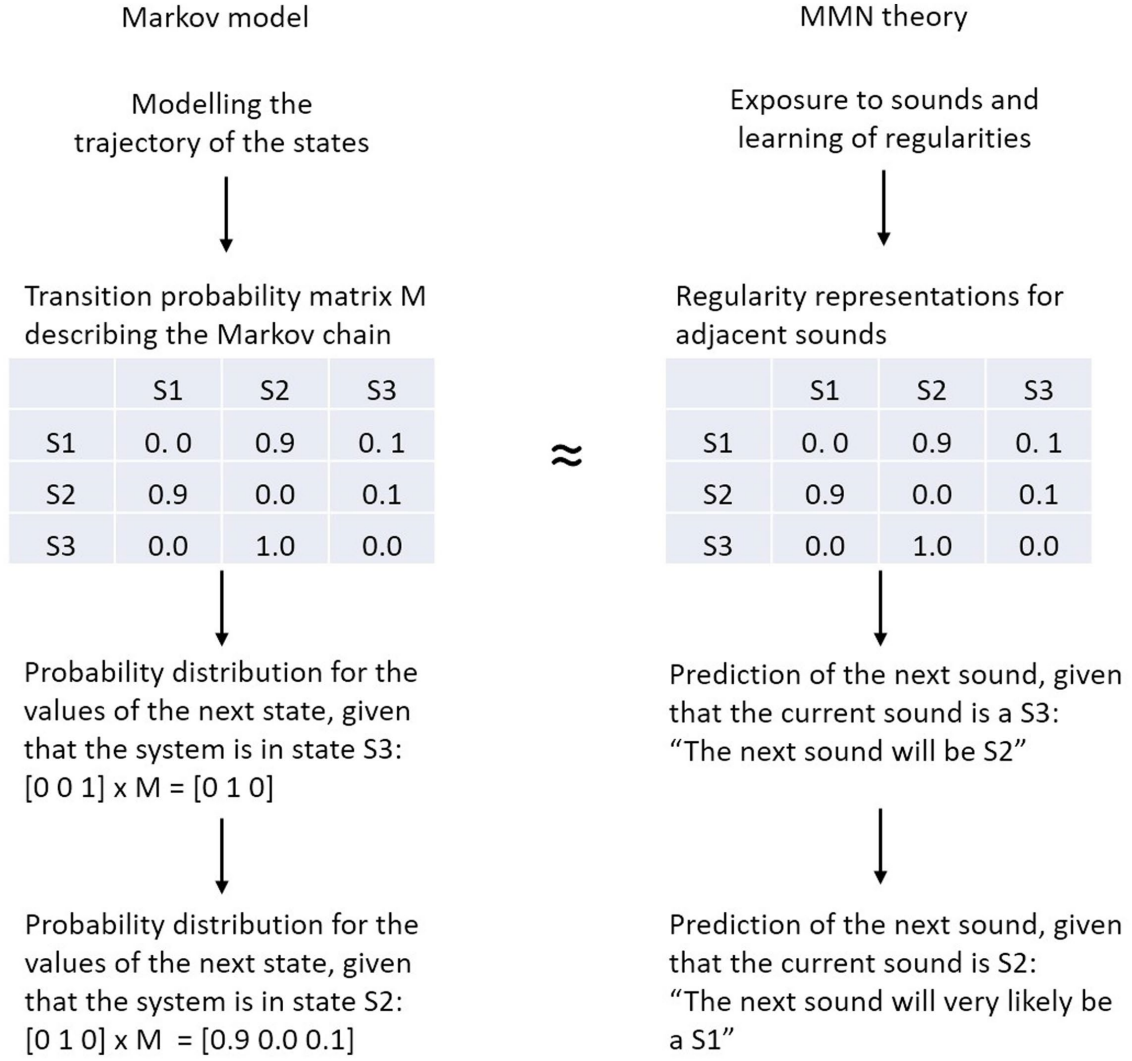
Analogy between the transition probability matrix of the Markov model (left) and the predictive regularity representations of the MMN theory (right). In a Markov model, the next state of the system is computed by multiplying the current vector state with the transition probability matrix, in MMN theory the next sound is predicted on the basis of the detected regularities.

We explore whether and to what extent the transition probability matrix in the Markov model can be regarded as a generative model that, literally, generates (predicts) the next sound in the sequence according to the stochastic process defined by that model. In this Markovian narrative the transition probability matrix of a Markov chain serves as a proxy of the generative neural model (i.e., the predictive regularity representations) underlying MMN elicitation. Therefore, the regularities detected by the brain’s information processing system correspond to (parts of) the entries of the transition probability matrix (Figure 2). Of course, this is a simplification with respect to the actual implementation as generative models include predictions at different time scales and at different levels of abstraction, even for simple perceptual entities such as pitch (e.g., Balaguer-Ballester et al., 2009).

There is no possibility for the MMN-system to know what all possible states and their transition probabilities might be. Thus, the memory underlying MMN most likely only covers actually encountered states and their respective transitions. Thus, not all possible states and/or transitions are necessarily known, such that a given empirical transition matrix only ever represents a limited excerpt from the world. Yet, the incompleteness and ambiguity in sensory information is a problem any perceptual model needs to take into account. And furthermore, it may not even be desirable for a mental model of the sensory environment to represent all potential states to achieve sufficient predictive power as (memory) resources are limited. Thus, it is an interesting question which states and transitions are represented in the human brain—that is, to what extent our mental models may show Markovian properties. For instance, is it necessary to encounter a given state a certain number of times before it is included in the model? Fortunately, key assumptions made by Markov models can be translated into specific predictions, which can be tested empirically such as that precision trumps base rate (*cf.* chapter “Classical oddball paradigms”). Another difference between the Markov chain as a mathematical model and our brains is that we do not have perfect memory. Whereas all events contribute equally to the Markov model in theory, both decay and interference but also current goals can result in unequal weighting of the encountered evidence in human memory.

On that note, two forms of underlying memory have to be distinguished here: First, the memory which corresponds to what has been learned from the preceding event sequence, that is, the probabilities of the transitions between events, which correspond to entries in the transition matrix of the Markov chain model. Second, the memory of the windoe of the event sequence that is considered by the MMN system: its length corresponds to the order of the Markov chain model.

Please note that the characteristic of a Markov chain being memoryless (Markov property, see above) and the assumption that the MMN-system is based on memory are not contradictory. This is, because the entries in the transition probability matrix represent the accumulated information about the succession of the different events (set of states in Markov terminology) over time. The transition probability matrix contains the previously encountered transitions (association of events) between the states at trial n-1 and trial n. Thus, we regard the transition probability matrix as a form of associative memory. More specifically, it is a directed association, because transition probability is specific to the order of two states (i.e., the transition probability from A to B is not the same as from B to A). Please note that the trajectory of experiences resulting in a specific memory content is not necessarily part of the memory itself. For instance, in other forms of implicit or explicit memory, such as declarative knowledge as in “Paris is the capital of France,” it is not uncommon that we do not remember how we obtained that piece of knowledge in the first place.

The transition probability matrix summarizes what *has been* the case in the past, in order to enable rational guesses about what *will b*e the case in the future. To elaborate, when taking the situation described by the Markov model in Figure 2 (i.e., the transition probability matrix), the MMN-system would make, metaphorically spoken, inferences like this: “The current sound is S3, from the entries in the respective row of the transition matrix it follows that the next sound will be S2” or “The current sound is S2, from the entries in the respective row of the transition matrix it follows that the next sound will most likely be S1.” Note, in the brain this process may not be realized in the form of explicit inference, but rather in the form of a joint representation of highly-associated stimulus features. This may be the case because the occurrence of parts of the constituents of a given object are already sufficient to activate the object’s full representation (Gregory, 1980)—a phenomenon described both in the concept of predictive regularity representation in MMN research (Winkler and Schröger, 2015) and in general principles of the representational brain such as in the theory of event coding (Hommel et al., 2001).

We are aware that the Markovian narrative does not necessarily directly lead to an adequate understanding of the specific neuroanatomical or chronometric structure of the MMN system, but it draws attention to a central aspect of theories of MMN—namely the encoding of transitions between sounds. Whatever the exact nature of the MMN system is, eventually a theory of the MMN system has to come up with an explanation for the presence or absence of differences in brain activity (or behavioral performance) in response to events differing in conditional probability. We propose that the Markovian view can aid to formulate and to test constraints of MMN theory. For example, theoretically relevant transitions between sounds can be accessed as cells or combinations of cells in the transition probability matrices. We argue that such an approach may stimulate MMN experiments and even lead to reinterpretation of existing research findings. In the following, we will examine three types of MMN paradigms from the Markovian view:

(1) Classical oddball paradigms emphasizing the different probabilities of different sounds types. The respective MMN theory postulates the importance of a memory encoding the features of the highly probable standard (i.e., a template of a specific stimulus). The Markovian view adds the potential importance of memory for transition probabilities between the different sound types to classical MMN theory and posits ways to test whether they contribute to MMN. Importantly, the Markovian view allows to dissociate between global probability (or base rate, i.e., how frequently is a specific state encountered) and local probability (or conditional probability, or precision, i.e., how probable is a specific state given the current state). We will elaborate on this distinction and how it can promote our understanding of the processes underlying MMN.

(2) Between-sound regularity paradigms that focus on transition probabilities between adjacent sounds. The respective MMN theory emphasizes the role of detected regularities in those transitions to be used in a predictive manner. Although this is a showcase for the Markovian view, there are implications that deserve experimental proof. One of the theory’s implications is that the frequency of the occurrence of the sound *per se* (event probability or base rate of the event) should not matter as it does in the classical MMN paradigms. Also, only the relative frequency of the occurrence of the transitions should matter, but not their absolute frequency. One or both of these assumptions might not be met. A resolution of these issues is of special interest with regard to advancing the dispute whether MMN serves as a comparator process or reflects adaptation. In addition, for between-sound regularity paradigms the Markovian view hints at asking which type of Markov chain is a reasonable proxy for the regularity representations underlying MMN. For example, some data appear to be compatible both with a first-order and with a second-order Markov chain, we will expand on this further on.

(3) Action-sound coupling paradigms use associations between button-presses and the sounds they produce. Strong associations (i.e., large transition probabilities) between a particular button-press and a particular sound are assumed to shape respective expectations of the sound following a button-press. Unlike in classical oddball paradigms and in between-sound regularity paradigms, predictions related to the next sound are not based on sound regularities, but rather on the association between an action and its (sound) effect. The intention to generate a particular sound (action-effect) includes the prediction that this particular action (e.g., left-button press) will yield the intended effect. In fact, according to ideomotor theory (James, 1890; Shin et al., 2010), the intended effect guides the selection of the respective action generating this effect. The Markovian view would suggest that these action-based predictions are not different from the predictions based on sound regularities. If this were so, these action-sound coupling paradigms are well suited to study MMN mechanisms because they avoid concurrent local and global sound probability effects because a rare action can be associated with a high probable action-effect (low global sound probability but high local sound probability).

## Distinction from previous Markovian approaches to MMN (and related research)

Regularity extraction, i.e., the learning processes underlying MMN elicitation include many facets. One of the facets is statistical learning, which is a diverse topic on its own that is covered by theories from cognitive psychology on various cognitive abilities in which the statistics of (co-)occurrences of events matter such as language acquisition (Saffran et al., 1996; Thiessen, 2017; Frost et al., 2019; Conway, 2020; Endress and Johnson, 2023). The Markovian view on the MMN we discuss in the present paper naturally is a simplification and it disregards many aspects of statistical learning.

Moreover, there exist computational theories that consider the emergence, maintenance, updating of and the competition between predictive models for sound sequences (Denham and Winkler, 2006; Meyniel et al., 2016). For example, in their paper, Meyniel et al. (2016) elaborate on the hypothesis that the brain continuously updates the time-varying matrix of transition probabilities between the stimuli it receives. Harrison et al. (2020) presented a variable-order Markov model which can learn higher-order statistics that change over time and which can consider memory constraints in detecting recurring tonal patterns. Unlike the present Markovian perspective, some computational theories take into account the neuroanatomical underpinnings of predictive models (Friston, 2005; Kiebel et al., 2009; Wacongne et al., 2012; Maheu et al., 2019; Tabas, 2021; Chien et al., 2022). In a very recent computational approach tailored to model predictability of sound sequences, Chien et al. (2020) the simulated signals for predictable and unpredictable sound sequences resembled the observed MEG amplitude traces from a study by Barascud et al. (2016). Their model included short term plasticity for the neural responsiveness not only for the single tones, but also for the combination of successive tones, thus considering both, event probability and transition probability. Another important paper by Mill et al. (2011) shows that a Markov chain of two states is useful to disentangle effects of probability (frequent standard versus rare deviant) from effects of switching (from one sound to another; namely, from standard to deviant) in oddball sequences. With their computational model based on the convergence of depressing synapses at the single neuron level, plenty of SSA effects can be simulated.

As mentioned above, we know of only a few MMN studies that explicitly refer to a relation between the MMN-system and Markov chains or transition probabilities (Furl et al., 2011; Koelsch et al., 2016; Mittag et al., 2016; Tsogli et al., 2019, 2022; Korka et al., 2021). In the study by Furl et al. (2011), participants learned second-order Markov sequences of pure tones (with unpredictable first-order transitions): that is a certain succession of two tones (a pair) predicted with high probability which of five tones would follow next. Improbable compared to probable transitions evoked increased MEG responses 150–200 ms after tone onset reflecting higher-order statistical learning. Koelsch and colleagues (Koelsch et al., 2016; Tsogli et al., 2019, 2022) presented isochronous sequences of sound triplets (characterized by timbre) to their participants in several studies. The first two sounds in such a triplet were fixed, while the probability of the third one varied. Low probability endings elicited an early anterior negativity that had an onset around 100 ms. Hence, Koelsch et al. observed the characteristic frontocentral negative deflection in the MMN range and suggested that this reflects statistical learning of transitional probability distributions. Mittag et al. (2016) tested whether MMN relies on probabilities of sound patterns or on transitional probabilities by presenting rare tone-triplets among frequent standard triplets. Results showed that deviance detection underlying MMN uses transitional probabilities.

Taken together, these empirical papers demonstrate the relevance of between-sound transitions and have thus improved our understanding of the MMN-system. Furthermore, these findings are well compatible with a Markovian view. In extension to these previous papers, in the following we elaborate on how a Markovian approach can be used for MMN research in a more systematic, canonical manner. To our knowledge, this is the first review on the relation between Markov models and MMN theory. However, the present paper only covers part of this relation. It disregards the development and updating of the predictive model itself. Rather, we explore what the predictive model underlying MMN looks like when interpreting existing data under the Markov narrative, thereby making some of the assumptions about the MMN-system explicit and pondering how those could be tested experimentally.

## Classical oddball paradigms

In the classical oddball paradigm one tone (e.g., a high-pitch tone), the standard, is presented frequently (simple repetition rule) and another tone (e.g., a low-pitch tone), the deviant or oddball, is presented rarely; for example, p(Standard) = 0.9 and p(deviant) = 1- p(Standard) = 0.1. Deviants elicit the MMN (mismatch hypothesis) and/or an enlarged N1 (or smaller P2) compared to the adapted N1 (or P2) for standards (∆N1; adaptation hypothesis). This classical oddball paradigm has been used in a plethora of MMN studies for various research questions. The elicitation of MMN is interpreted as evidence that the deviant has been detected by the information processing system and that some form of representation of the standard (and/or the regularity) had been established. As MMN is elicited even when participants do not intend to detect deviants or when they do not actively attend to the sound sequence, there is common agreement that MMN taps into implicit memory functions of the brain that govern subsequent, more conscious information processing (Tiitinen et al., 1994; Näätänen et al., 2011).

Classical MMN research shows that all basic dimensions of simple sounds such as pitch, location, duration and loudness are included to the memory representation underlying MMN. Moreover, also complex dimensions of sounds such as timbre, harmonicity or broadness are represented without explicit intention, as are speech signals (e.g., phonemes) and other natural sounds (e.g., ringtones). Many studies determined the influence of attention on the respective MMN, or compared MMN between different age groups or clinical groups for a range of features. Others were interested in the acquisition and maintenance of the memory of the representations involved in mismatch processing and the influence of other cognitive factors such as attention or familiarity with the sounds (Näätänen, 1988, 1992; Bartha-Doering et al., 2015; Kirihara et al., 2020; Tervaniemi, 2022; Weise et al., 2023).

**Figure 3:**
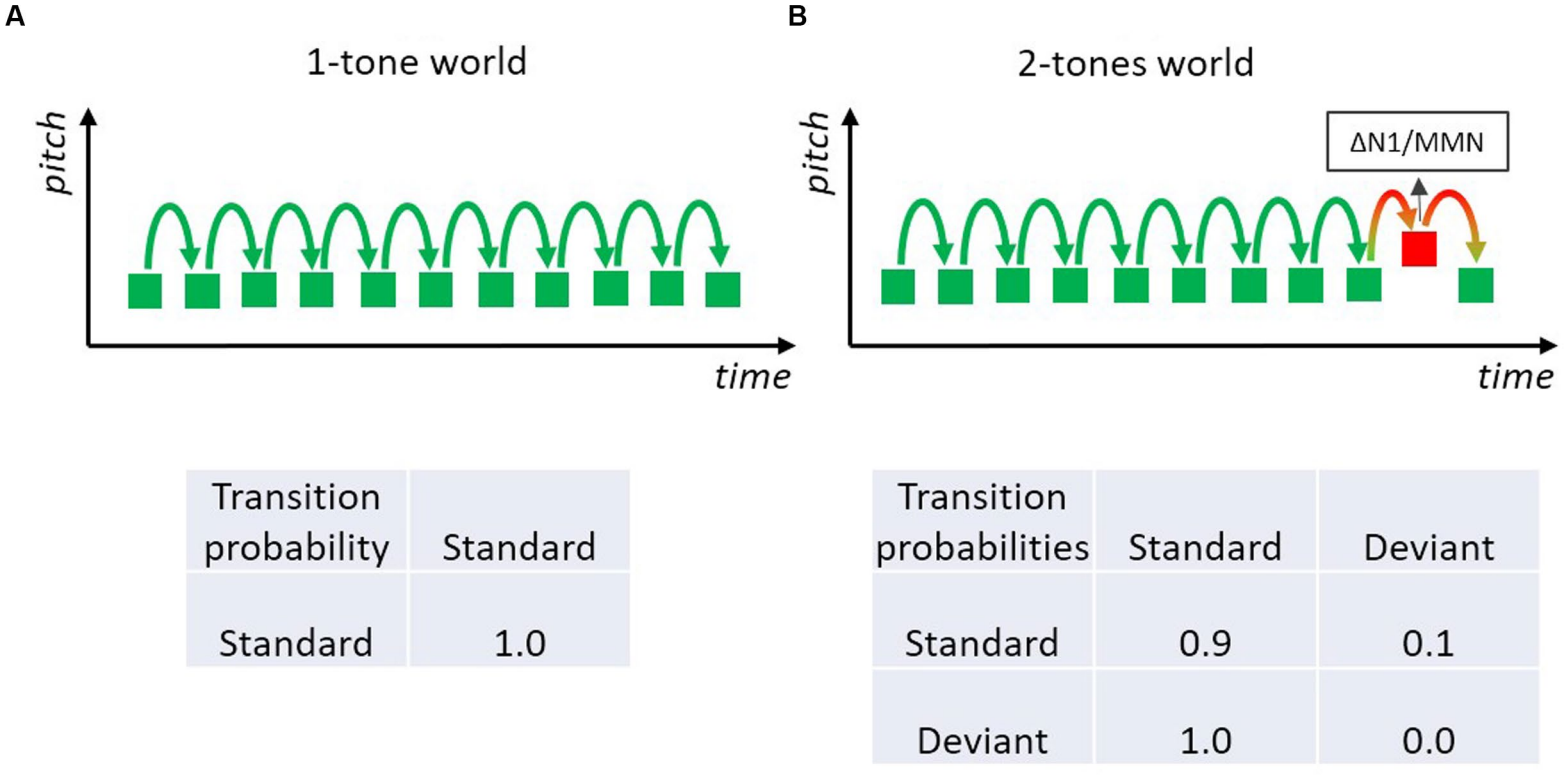
Two-tone classical oddball paradigm with a frequent standard (green) and an infrequent deviant (red) tone. Deviants elicit the MMN, which may be regarded as a genuine mismatch (prediction error) signal and/or the expression of differential adaptation to feature-specific neurons underlying the N1 (resulting in a small standard-N1 and a large deviant-N1). **(A)** Only frequently presented standard sounds are regarded as constituting the rule in the tone sequence; so only one type of transition has to be modeled; the respective transition probability matrix specifying the one transition is shown below. **(B)** The stochastic process of the two-tone sequence is characterized by a 2-state Markov model with four transitions (three are actually shown): each could be of possible relevance for the experiment; the respective transition probability matrix specifies the four possible transitions.

There are many ways to “translate” an oddball sequence of tones into a Markov chain. The simplest way is to pretend that there is only one rule which is defined by the repeated presentation of the standard tone (Figure 3A). In this case, the probability of the standard tone and also the transitional probability that a standard is followed by a standard is 1.0. If the sequence is modeled by the repetition rule, the deviant only “serves” as a challenge to this one-standard world, in order to test whether the rule has been encoded and whether a mismatch will be registered when an event does not conform to the rule. In fact, this is the prevailing description of an MMN experiment, likely because MMN researchers usually focus on the rules defined by the frequent standard. However, the sequence of sounds in these MMN experiments actually corresponds to a stochastic sequence including both standard and deviant sounds. Especially, when considering that typically deviants occur with some 10–20% probability, such that about every tenth to every fifth tone is a deviant. Therefore, a Markov chain that includes both standards and deviants is arguably more adequate to model an MMN-experiment than one that includes only the most frequent sound.

A Markovian look at the transition probability matrix in a sequence with p(Standard) = 0.9 and p(Deviant) = 0.1 immediately reveals that there are two transitions with rather high probability values: the transition standard-to-standard with a value of 0.9 and the transition deviant-to-standard with a value of 1.0 (Figure 3B). The latter is because there are intentionally no transitions from deviant to deviant in many MMN experiments. Consequently, the strongest rule (in terms of precision) in the typical MMN experiment is not that a standard follows a standard, but rather that a standard follows a deviant. Hence it is of high theoretical interest whether violations of this rule matter. If they do, the notion that transition probabilities between events are of importance (not merely the event probabilities, i.e., their base rate) would receive strong support. As the Markovian perspective predicts that precision (how probable is a specific transition) is more important than the base rate of transitions (how often is a specific transition encountered), this example raises the question whether highly probable transitions are learned even when that transition is encountered only occasionally, as is the case with deviant-to-standard transitions. Nevertheless, the view that only-standards define the rules is so dominant that many researchers using classical MMN experiments (including those of our group) usually do not pay attention to transitions from rare, deviant sounds even if those transitions are of high probability.

**Figure 4:**
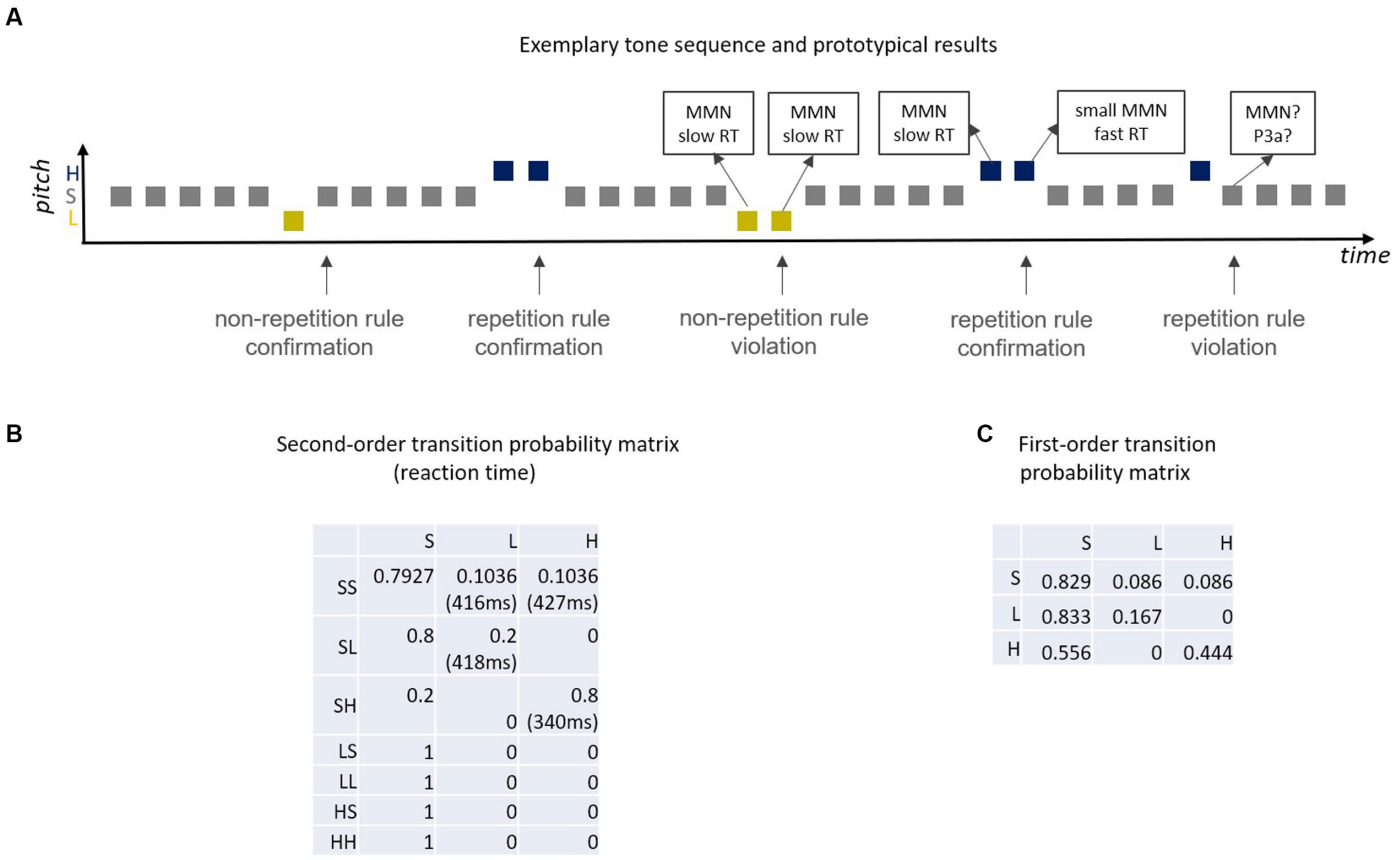
**(A)** Exemplary sound sequence of Coy et al. (2022) and prototypical results [MMN, RT (reaction time)]; unpredictable deviants elicit full amplitude MMN, predictable deviants smaller MMN (e.g., Sussman and Winkler, 2001) and, when deviants are targets, shortened RT (*cf.* Coy et al., 2022); according to the Markovian view, standards that create a deviant-repetition rule violation should elicit MMN and/or P3a. **(B)** Second-order transition probability matrix (S = standard, L = Low pitch deviant, H = High pitch deviant; SL = standard followed by Low deviant, …); e.g., cell(1,2) denotes the probability (0.1036) that an SS pair is followed by an L sound (low deviant); reaction times for deviants are listed in brackets; second deviants with high transition probability [i.e., HH, cell(3,3)] have by far the shortest reaction time. **(C)** First-order Markov chain that generates a highly similar tone sequence to the second-order Markoc chain in **(B)** but which represents a rather different proxy for the predictive memory underlying MMN.

Many classical MMN studies can be described by transition matrices similar to the one in Figure 3B. However, as mentioned above, the majority of these studies focused only on the base rates of the two possible events. This view only considers brain responses to events that resulted from two out of the four possible transitions—namely the transition from a standard to a standard and from a standard to a deviant. The standards in deviant-to-standard transitions were mostly neglected (excluded from analysis) and those from deviant to deviant largely do not occur within such sequences. The latter is due to an additional constraint in the design in most oddball experiments: each deviant has to be followed by at least one standard, which means that the deviant-to-standard transition probability is 1.0 (and the deviant-to-deviant transition is 0.0). If transitions matter, effects on brain and/or behavioral responses to violations of regular deviant-to-standard and deviant-to-deviant transitions should yield respective predictive regularity representations. Indeed, evidence for that was reported by Sussman and Winkler (2001). They were interested in the attenuation of the MMN to a deviant following a deviant (i.e., when a deviant is encountered after a sequence of standards a second deviant is presented with a certain probability before the sequence returns to the standard regularity). For that purpose, they manipulated the deviant-to-deviant transition probability, that is the conditional probability of deviant repetition (similar as in Figure 4A). Indeed, they found a stronger MMN reduction for the second deviant, if a deviant repetition is more likely to occur. In fact, there exist some studies reporting similar MMN attenuation for the second deviant relative to the first deviant (Sams et al., 1984; Deacon et al., 2000; Müller et al., 2005; Berti, 2008; Todd and Mullens, 2011).

However, one should consider that there is a different amount of SSA in standard-standard-*deviant* (first deviant) and standard-deviant-*deviant* (second deviant) sequences (Ulanovsky et al., 2004). Thus, the comparison of first and second deviants is not well suited to measure the decline in MMN as an indicator for predictability. Lecaignard et al. (2015) controlled for adaptation effects in their study in which they compared ERPs for predictable and unpredictable deviants. The authors reported a decrease of the MMN with predictability. Interestingly, a similar effect occurred both at earlier latencies (70 ms after deviance onset) and at later latencies (300 ms, P3a).

Importantly, to our knowledge in none of the studies presenting successive deviants MMN was fully absent for a second deviant with high conditional probability. However, as mentioned in the previous paragraph, in almost all these studies second deviants are not compared with respective low repetition probability deviants but with first deviants (which are preceded by a standard). That is, possible adaptation effects are not controlled for. Moreover, to our knowledge, it has not yet been tested whether a standard unexpectedly following a first deviant (Figure 4A) yields a prediction error signal (MMN or P3a). If the MMN were only about transition probabilities, both the absence of a high-probable second deviant MMN and the presence of a fully-fletched MMN for standards following a first deviant that usually is followed by a second deviant would be expected. The finding that sound probability matters, questions the hypothesis that MMN solely relies on transition probabilities and is compatible with the adaptation hypothesis explaining (at least a part of) MMN as ∆N1 and/or ∆P2. However, the question still remains whether the conditional probabilities for stimuli following a deviant do or do not matter for the predictive model. To address this question, Coy et al. (2022) recently developed a modified oddball paradigm that improves control of confounds inherent to these previous studies.

In the Coy-paradigm (Figure 4A), there are two different pitch deviants. One of them is associated with a high transitional probability to a standard (non-repetition rule: yellow deviants). The other pitch deviant is associated with a high transition probability to a deviant of the same pitch (repetition rule: purple deviants). With this manipulation Coy et al. embedded two diametrically opposed repetition rules in the stimulation sequence: when encountering a first deviant, the deviant type (i.e., the tone’s pitch) allows to predict whether the next sound is either more likely a standard or more likely another deviant. In a subset of cases these deviant-type specific transition rules are “violated,” that is, the non-repetition rule deviant repeats, the repetition rule deviant does not. In a proof-of-principle behavioral study, Coy et al. (2022) asked participants to press a button in response to all high and low-pitched sounds (i.e., all deviants), but did not inform them that the transition probabilities to the tone following a first deviant (which could be a deviant or a standard) were manipulated. Although participants were naïve to the repetition rules, detection speed was facilitated when a second deviant was predictable (high transitional deviant-to-deviant probability) but not when a second deviant was unpredictable (low transitional deviant-to-deviant probability). In the case that a first deviant was followed by a standard, the rate of false alarms increased when this transition was improbable (low transitional deviant-to-standard probability, i.e., high transitional deviant-to-deviant probability) compared to probable (high transitional deviant-to-standard probability, i.e., low transitional deviant-to-deviant probability). This paradigm has the advantage of including a reversed control condition for any chosen conditional deviant repetition probability so that the comparison between confirmation and violation of both a repetition rule (as in deviant repetition paradigms) and a non-repetition rule (as in the classical oddball paradigm) is enabled. Importantly, this paradigm provides a framework to dissociate local rules (defined by transition probability) and global rules (defined by base rate of stimuli or transitions). Namely, deviants always violate the global regularity because they are rare by definition, whereas standard always conform to the global regularity because they are frequent. Thus, within this paradigm one can compare whether the sound following a first deviant is improbable or probable based on the transition probability (high vs. low) associated with the preceding stimulus both when the sound in question is rare (deviant) or frequent (standard).

Please note, that in this paradigm the probability of the next tone does not only depend on the current tone, but also on the previous tone. Stochastic sequences in which the probability of a future state is based on the two previous states (second-order dependencies) can be modeled as second-order Markov chains (Figure 4B). An interesting comparison in this matrix is between reaction times of the last tones in the SS-**H** and the SH-**H** sequence with respective transition probabilities of 0.10 [cell(1,3) in Figure 4B] and 0.80 [cell(3,3) in Figure 4B]. With this the differences in reaction times for a first high deviant (low transition probability) and a second (repeated) high deviant (high transition probability) can be assessed. Another interesting comparison is between SL-**L** [cell(2,2) in Figure 4B] and SH-**H** [cell(3,3) in Figure 4B], that is, between a second low deviant (low transition probability: 0.2) and a second high deviant (high transition probability: 0.8). These two comparisons yield reaction time advantages of 84 ms (first against second H deviant) and 78 ms (L against H second deviant) for the high-probability repeating deviant, demonstrating that high probability of deviant repetition manifests as a huge decrease in reaction times. Presumably, this is because a high compared to a low transition probability is equivalent to a better predictability of this sound.

However, it should also be noted that the sound sequence in this study cannot only be modeled by a second-order Markov chain, but—although less accurate—by a first-order Markov chain (only considering transitions between adjacent sounds). In fact, the respective first-order Markov chain (Figure 4C) yields highly similar sequences as compared with the sequences for the second-order Markov chain. HH successive deviants have a much higher transition probability than LL successive deviants in the first-order Markov chain (0.44 vs. 0.17). Thus, one cannot be sure whether the actual difference in reaction time between second deviants in LL and HH pairs of Coy et al. (2022) study is based on a first- or on a second-order Markov model. Such a reinterpretation of the study’s data results in a different, more parsimonious conceptualization of the underlying generative model for the information processing system: the brain considers transitions between adjacent sounds only (first-order)—as opposed to the brain considers transitions between adjacent sound pairs (second-order). In order to decide, which alternative better explains the data, one could perform a model comparison and/or design an experiment including critical conditions discriminating between the two alternatives. One potential approach could be to play short excerpts from these sound sequences and ask participants to decide which sound should follow the last sound they heard. Their response can be compared with the respective prediction inferred from the first- and from the second-order Markov model. In any case, the Coy et al. (2022) study provides evidence that transition probabilities matter for the performance in a behavioral task. Due to the nature of the task (only demanding a behavioral response to the deviants) other interesting comparisons between cells were not possible. For example, comparing cells with equal transition probability but different base rate probability (and vice versa) would be interesting [e.g., SH-S cell (3,1)] and SL-L cell [(2,2) in Figure 4B]. Also, the comparison of pairs of cells with equal ratio of transition probabilities, but largely different transition probabilities *per se* could be tested in future studies. For example, consider a scenario in which the transition probability for event B preceded by event A is twice as large as for D preceded by C (ratio of transition probabilities equals 2). One might expect processing benefits for event B as compared to event D, as the relative predictability is higher. However, an identical ratio of transition probabilities can be achieved with two small transition probabilities (e.g., 0.2/0.1 = 2), but also with one large and one small transition probability (e.g., 0.8/0.4 = 2). This may or may not make a difference in the brain’s predictive processing.

To conclude this section, it should be mentioned that it is also possible that the brain may not necessarily operate on a strictly first-order or strictly second-order Markov chain, but that the order of the model (i.e., how many preceding states are considered for a current prediction) may be dynamic (Harrison et al., 2020).

## Between-sound regularity paradigms

**Figure 5:**
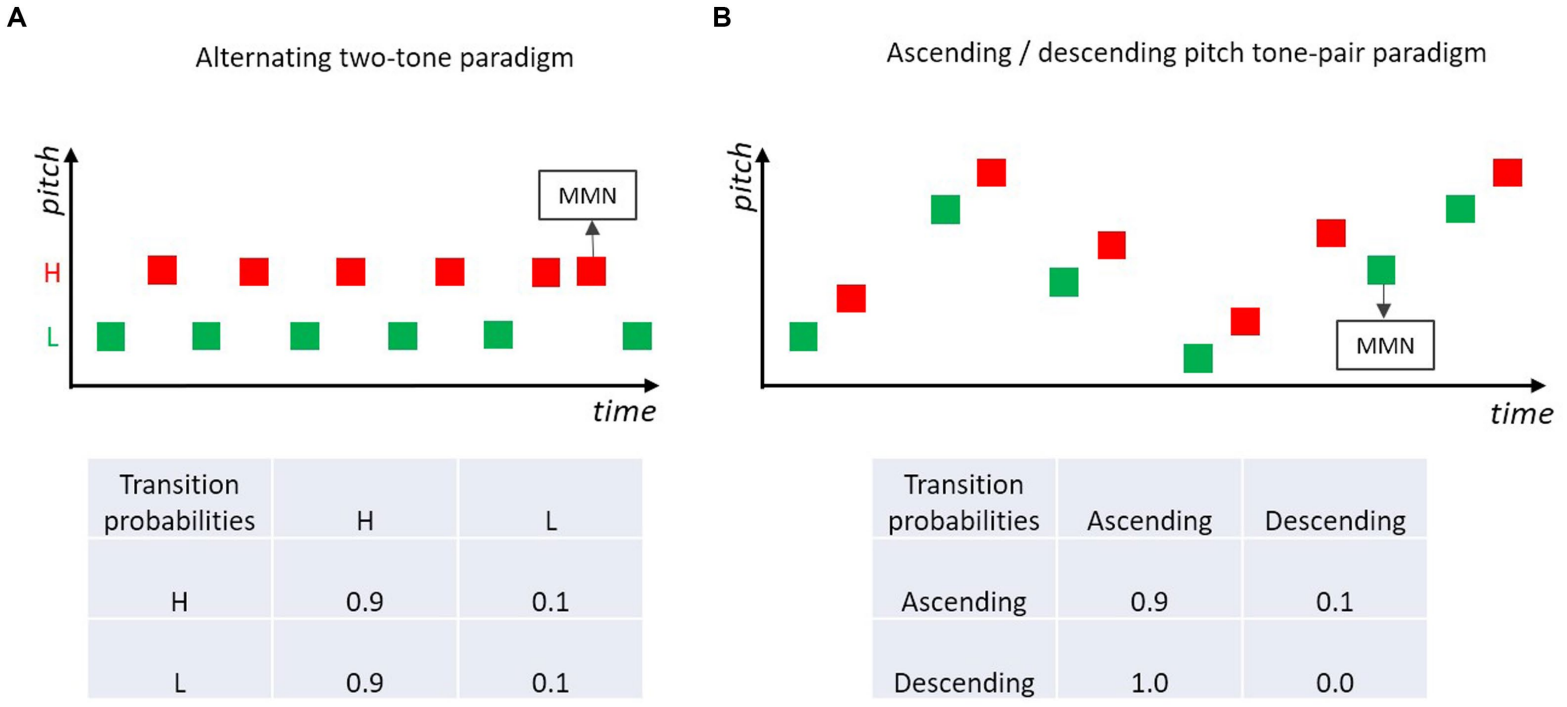
**(A)** Two-tone alternating paradigm, where a low and a high pitch tone are presented alternatingly, occasionally a high- or a low-pitch tone is repeated. Violations of the alternation rule usually elicit MMN (e.g., Nordby et al., 1988; Alain et al., 1994; Winkler and Czigler, 1998; Horváth et al., 2001). **(B)** Tone pairs are presented in ascending (high transition probability) or descending (low transition probability) pitch. Violations of the prevailing rule elicit MMN (e.g., Saarinen et al., 1992; Paavilainen et al., 1999).

A showcase for the hypothesis that MMN is based on the encoded relations between successive sounds, is a paradigm in which two (or more) different tones are structured as a regular sequence. For example, in a sequence of two alternating tones (e.g., high-pitch tone, low-pitch tone, high-pitch tone, low-pitch tone, …; Figure 5A) occasional order reversals of the two tones (i.e., the repetition of a given sound) elicit MMN (Nordby et al., 1988; Alain et al., 1994; Winkler and Czigler, 1998; Horváth et al., 2001). In this type of paradigm, adjacent tones conforming to the alternation rule have high transition probability, tones violating the alternation rule (i.e., tone repetitions) have low transition probability. MMN is elicited by violations of the order rule, even when the base probability of the irregular sound (violating the order rule) is the same as the base probability of the rule conforming sounds.

An interesting variant of this paradigm has been introduced by Winkler et al. (2012), Mill et al. (2013), and Bendixen (2014) to study the streaming or grouping of the tones. They presented triplets of sounds in a fixed order with occasional deviations breaking this order. One instance of this is the so-called galloping paradigm (ABAABAABA): this sequence can be heard as a single stream comprised of ABA_ABA_ABA triplets (sounding like a galloping horse, hence the name) or as two separate streams of single tones—namely, an A-stream and a B-stream. The percept can switch between the two alternatives (grouping versus streaming) as a function of the frequency separation, the length of the silent interval between the tones, and the intention of the listener. In the grouping case, the respective predictive regularity representation can be conceptualized as a Markov chain with the possible standard state being ABA, whereas in the streaming case, the predictive regularity representation can be described as two separate Markov chains. A computational model for simulating such possible perceptual organizations (and their underlying predictive representations indicated by MMN) is CHAINS (Mill et al., 2013).

This between-sound regularity paradigm illustrates that an input sequence can usually be “tokenized” in more than one way. The respective tokens correspond to the events (states) in the Markov chain, potentially used by the MMN system. Naturally, not all of the possible tokenizations are equally successful in terms of increasing the predictability of the sequence. When assuming that the goal of the brain is to maximize predictability of the sequence, identifying Markov chains with states (tokens) revealing high transition probabilities certainly aids in achieving this goal. This may be one mechanism through which perceptual organization (i.e., model selection) is driven.

The Markov view is very simplistic, and lacks a specification of both the mechanism of how the sources of a sensory input are identified and how they are implemented in the Markov model. There exist more advanced approaches to tackle this question, for example in an evidence accumulation model by Barniv and Nelken (2015) that explains streaming, or in a more general Bayesian causal inference model by Shams and Beierholm (2022) that determines (competing) causal structures and evaluates them—such approaches generalize beyond auditory streaming/grouping phenomena.

Instead of isochronous presentations of single tones, tonal patterns consisting of concatenated tones with constant transitions between the tonal elements have been used in many MMN studies. For example, pairs of two tones differing in frequency (1,000 vs. 1,500 Hz) were presented in a study by Tervaniemi et al. (1999). In standard tone pairs (*p* = 0.9), the tones’ frequencies were in an ascending order (second tone is higher than first), whereas in the deviant pairs the tones’ frequencies were presented in descending order (second tone is lower than first). MMN was elicited in response to those deviant pairs. Such regularities defined by patterns can consist of more than two tonal elements and do not only pertain to transitions defined by pitch relations, but also to other features such as duration or intensity (Schröger et al., 1995; Winkler and Schröger, 1995). Interestingly, the absolute feature values defining the relation between the tones within a pattern (e.g., tone pair) do not need to be constant within a stimulation sequence (e.g., 1,000 vs. 1,500 Hz) but the standard regularity defined by within-pair pitch direction can also be extracted when absolute feature values randomly vary between trials along the dimension of interest (here, pitch) (Schröger et al., 2007). Figure 5B illustrates such an “higher-order change” in the relation between the two tones in a pair (Saarinen et al., 1992; Paavilainen et al., 1999; Schröger et al., 2007; Paavilainen, 2013). Violations of the dominant within-pair pitch relation elicit MMN. These MMN studies with frequent (absolute and relative) relations in some feature between paired/grouped tones show that the Markovian view is not only applicable to transitions between adjacent stimulus events consisting of a single tone, but can be generalized to transitions between stimulus events which themselves are defined by some transition rule (e.g., melodic or speech sound patterns). In other words, to some extent the processing principle underlying MMN can be regarded as recursive, potentially including several levels of increasing abstraction corresponding to the different levels of potential transitions within and between sound events.

It should be mentioned that the amplitude of MMN in response to violations of the order of sounds is often somewhat smaller than the MMN obtained in the classical oddball paradigm. In principle, this observation is compatible with the hypothesis that two separate deviance detection processes contribute to the classical oddball MMN, one encodes the probability of sounds, the other the probability of transitions between adjacent sounds. The former deviant detection process is believed to represent some form of neural adaptation to the high probable standard (see above), which has been investigated in different contexts in human and animal research. It expresses as SSA to the regular sound in the neural firing rates in various species (for reviews Nelken, 2014; Willmore and King, 2023), in the amplitude of human mid-latency ERPs (for review see Grimm and Escera, 2012), the N1 ERP (Näätänen and Picton, 1987), and the P2 ERP (Haenschel et al., 2005). The latter deviant detection process is often described as a genuine mismatch response to the irregular sound, which cannot that easily be explained by a difference in neural adaptation to particular features of the regular and the irregular sound (Näätänen et al., 2005). However, although the auditory cortex consists of functionally distinct fields, it includes many interconnections (Kaas and Hackett, 1998). Thus, adaptation is not necessarily confined to local, channel-specific phenomena only. Rather, as explained by a model of May and Tiitinen (2010), the auditory cortex can be regarded as a system with variability in stimulus selectivity across cortical fields, resulting in complex and partially overlapping adaptivity patterns. Moreover, this system reveals a high context-dependence, which is achieved by (lateral) short-term synaptic depression. Newer variants of the May and Tiitinen model (Hajizadeh et al., 2019; May, 2021) are even able to model MMN in experimental scenarios that were previously taken as evidence against an adaptation account of MMN. For example, MMN to rare omissions of a sound (Yabe et al., 1998) or the MMN obtained in the multi-standard-paradigm controlling for first-order adaptation effects (Schröger and Wolff, 1996; Jacobsen and Schröger, 2001) can be simulated by the adaptation model of May (2021).

Within the framework of first- (feature values of the sounds themselves) and second-order (transitions between sounds) adaptation, smaller MMN in between-sound regularity paradigms compared to classical oddball paradigms can be explained as follows: In the latter paradigm both effects add up, whereas in the former paradigms, in which there is no one frequent standard sound but the between-sound transitions are constant, only MMN effects of second-order adaptation (or supposedly genuine MMN) emerge.

Within the adaptation framework, observed MMN can be attributed to a combination of adaptation both to feature values of the sounds themselves and to feature values of transitions between sounds (e.g., direction of pitch change). It seems possible that there is stronger adaptation of the neuronal populations specific to the standard in the classical oddball (because only two pitch values occur, one of which more frequently than the other) compared to a standard in a highly variable pitch-pair context (many pitch values occur with more or less equal probability), implying that there is less adaptation to a specific feature value. However, both in the classical oddball and in between-sound regularity paradigms the between-sound transitions occur frequently and consistently adapt the same neuronal populations. Thus, in between-sound regularity paradigms observed MMN is foremost driven by neuronal populations that adapt to frequently encountered transitions (smaller MMN), whereas in the classical oddball there is additional adaptation from the stimulus-features (larger MMN). Therefore, this could mean that instead of two adaptation mechanisms, there is simply a differential involvement of areas in auditory cortex that depends on the stimulus material/context employed in the study.

The newer computational models of adaptation phenomena and the latter considerations presented in the two previous paragraphs show how powerful neural adaptation mechanisms can be. This illustrates that interpreting predictive regularity representations of the MMN system as cells of the transition matrix of a Markov chain (see section “The Markov model as a proxy for the neural model underlying MMN”) is somewhat metaphorical. That is, the MMN system behaves as if it were a Markov model. The neural excitability in response to a predictable event may “simply” be reduced through adaptation processes, even when the transitions (i.e., the standard regularity) in question are rather complex. Under this assumption, the probabilities in the transition matrix represent the level of adaptation to a particular transition. This implies that (at least) the auditory cortex may possibly not make genuine *ex ante* predictions. Although, on a more cognitive level adaptation may be considered a mechanism of prediction nonetheless, following a simple heuristic—namely, “a transition that has been frequent in the past, is likely to persist in the immediate future.” That is, sensory prediction may correspond to optimizing neural processing to (i.e., to prepare for) events of high transition probability. In this sense, ‘predictions’ are implicit in the differential adaptation patterns across the brain networks (Denham and Winkler, 2020).

Applying their generic deviance detection principle in a computational model, Chien et al. (2019) were able to simulate omission responses and MMN as activity of the same change detector mechanism. According to their model, these change detection processes take place locally in the auditory cortex in the form of reciprocal connections. These reciprocal connections are the source of MMN. The preceding regularity formation is achieved by short-term plasticity, and this short-term plasticity makes local excitatory-inhibitory circuits prone to be change detectors. MMN and omission responses cannot be explained by sustained activity (resonance) caused by previous stimulation extending to the current trial, but rather correspond to genuine activity related to pre-activated responses at the time of the expected tone onset (Bendixen et al., 2009; Andreou et al., 2015).

Irrespective of whether one favors the powerful computational models of short-term plasticity in auditory cortex facilitating deviance detection via an emphasis on adaptation at local auditory levels (e.g., Mill et al., 2011; Chien et al., 2019; May, 2021), the predictive coding account of MMN emphasizing top-down predictive and bottom-up prediction error activity (e.g., Wacongne et al., 2012), or conceptual frameworks from cognitive psychophysiology locating the MMN between sensory and cognitive processing levels (e.g., Winkler and Schröger, 2015), there is plenty of evidence from experiments using between-sound regularity paradigms that transitions (between sounds) matter. Overall, this supports the Markovian view on MMN. However, this does not necessarily hold in any case. For example, Háden et al. (2023) reported that with temporarily jittered presentation of sounds newborn infants did not show statistical learning of transition probabilities.

**Figure 6:**
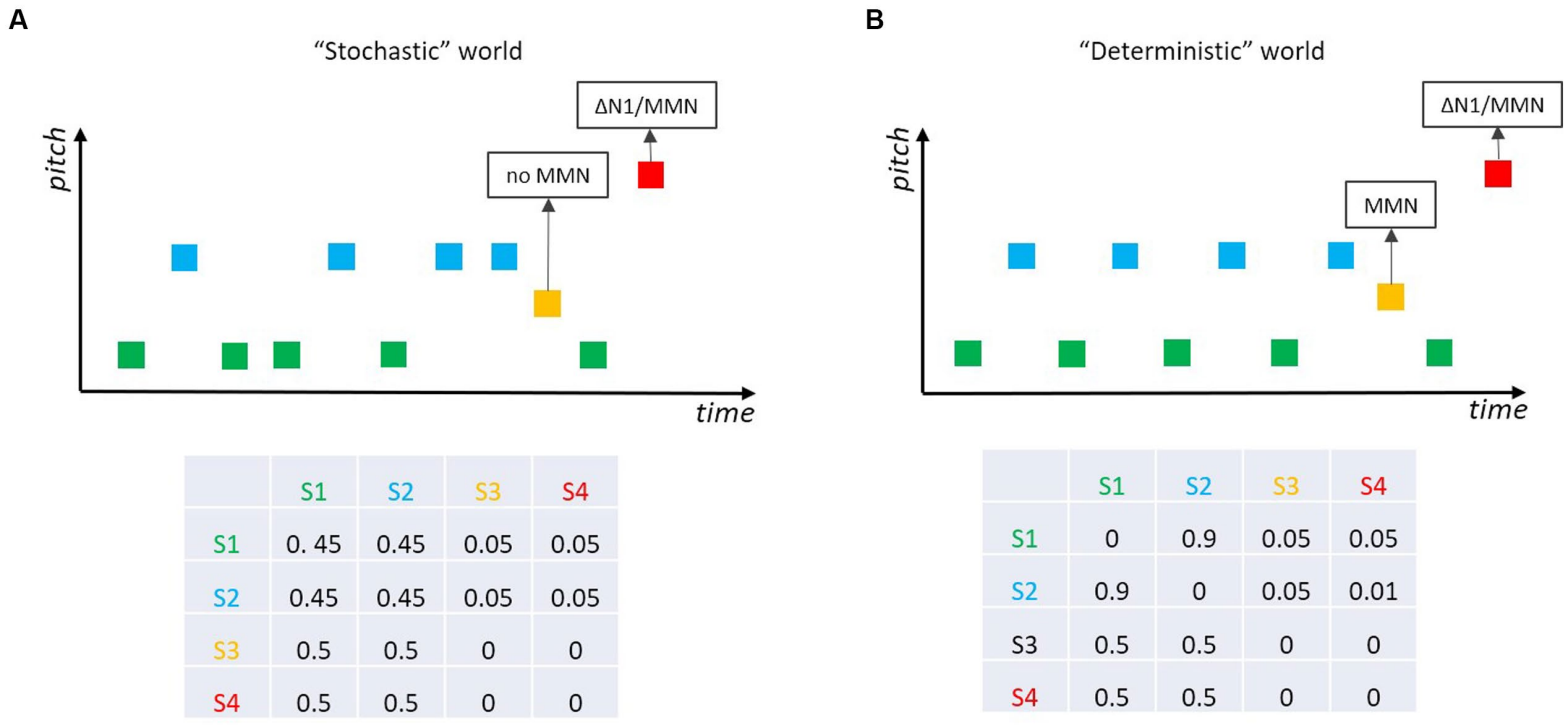
Two tones (green, blue) are played frequently, and two other tones (ocher, red) ralely. **(A)** In a “stochastic” world red tones elicit MMN/∆N1, while ocher tones do not as they belong to the distribution spanned by the standard tones (*cf.* Garrido et al., 2013; Schröger and Roeber, 2021). **(B)** In a “deterministic” world where the two standard tones ocher and blue alternate and become predictable, ocher (and red) tones elicit MMN; if the pitch separation between the two standard tones (green, blue) increases, deviant (ocher) tones elicit ∆N1 but still no MMN (*cf.* Schröger and Roeber, 2021).

One interesting sub-field of MMN research was motivated by the fact that our auditory world is not fully deterministic (as in classical MMN experiments), nor is it fully stochastic (as this would preclude the neuronal modeling of the world). Research by Winkler et al. (1990) and Garrido et al. (2013, 2016) revealed that the auditory system tolerates some stochastic variability in the statistical properties of incoming sounds. For example, Garrido et al. (2013) sampled the pitch of most of the tones (standards) from a Gaussian distribution, whereas “probe tones” were either equal to the mean of the distribution of the standard tones or largely outside this distribution. MMN was only elicited by tones outside the distribution (Figure 6A), with larger amplitude the smaller the variance of the Gaussian distribution for the standard tones was. Thus, the auditory system seems to be capable of learning non-deterministic, stochastic regularities from an uncertain world and of detecting outliers from a learned distribution. This has been an important step in MMN research as it reveals the ecological validity of the MMN approach. Yet, the distinction between deterministic and stochastic acoustic environments raises additional questions. Again, Markov chains are well suited to bring up interesting experimental scenarios contrasting mere “deterministic” with mere “stochastic” worlds. In a study by Schröger and Roeber (2021), the original finding in stochastic situations (Winkler et al., 1990; Garrido et al., 2013, 2016) was replicated (i.e., no MMN for tones at the center of the distribution of the standards; Figure 6A) and expanded to a partly deterministic world (Figure 6B), where the standard tones become predictable (e.g., because they follow an alternation rule). In this situation, deviant tones corresponding to the mean of the distribution of the standard tones elicit MMN, as they violate the prediction (regularity representation). The distinction between these two worlds (*cf.* Figure 6A vs. Figure 6B) represents an interesting scenario: it shows that the MMN system does not act as a human gambler would do—betting on green or blue in the stochastic world (Figure 6A). Please note, that also in the stochastic world, each of the two standard sounds is 10 times more likely to occur than a deviant sound (as they are in the deterministic world), and yet the MMN-system does not appear to generate respective predictions for the standards that will be mismatched when a deviant occurs. To our knowledge, it has not yet been systematically investigated when a stochastic world (e.g., in terms of ratios of the respective transition probabilities) becomes deterministic enough for the MMN system to get into action.

## Action-sound coupling paradigms

We do not only passively listen to sounds that happen in the acoustic environment, but we also intentionally generate sounds through our own actions, such as in speech and music or ringing a door bell. Oftentimes sounds provide feedback that a specific action achieved the desired outcome (or not), such as when hitting (or missing) a nail with a hammer. An increasing number of experiments used action-sound regularity paradigms to test for effects of violations or confirmations of action-effect intention. That is, whether or not an action yielded the intended, and thus predicted, effect. The results of these studies indicate that sounds conforming (relative to not conforming) to an intended action, and thus predicted effect, elicit N1 suppression (and MMN) in a similar way as sounds (not) conforming to an auditory rule (Rinne et al., 2001; Hughes et al., 2013; Korka et al., 2019, 2021; Darriba et al., 2021). Action-sound coupling paradigms illustrate that not only transitions from sound to sound can be relevant, but also transitions between actions and sounds. This makes these paradigms also an interesting target for our Markovian approach, considering that the Markovian perspective considers transitions between all possible states of a system, The respective transition probability matrices of such experiments can be easily constructed as action-to-sound and sound-to-action transitions.

**Figure 7:**
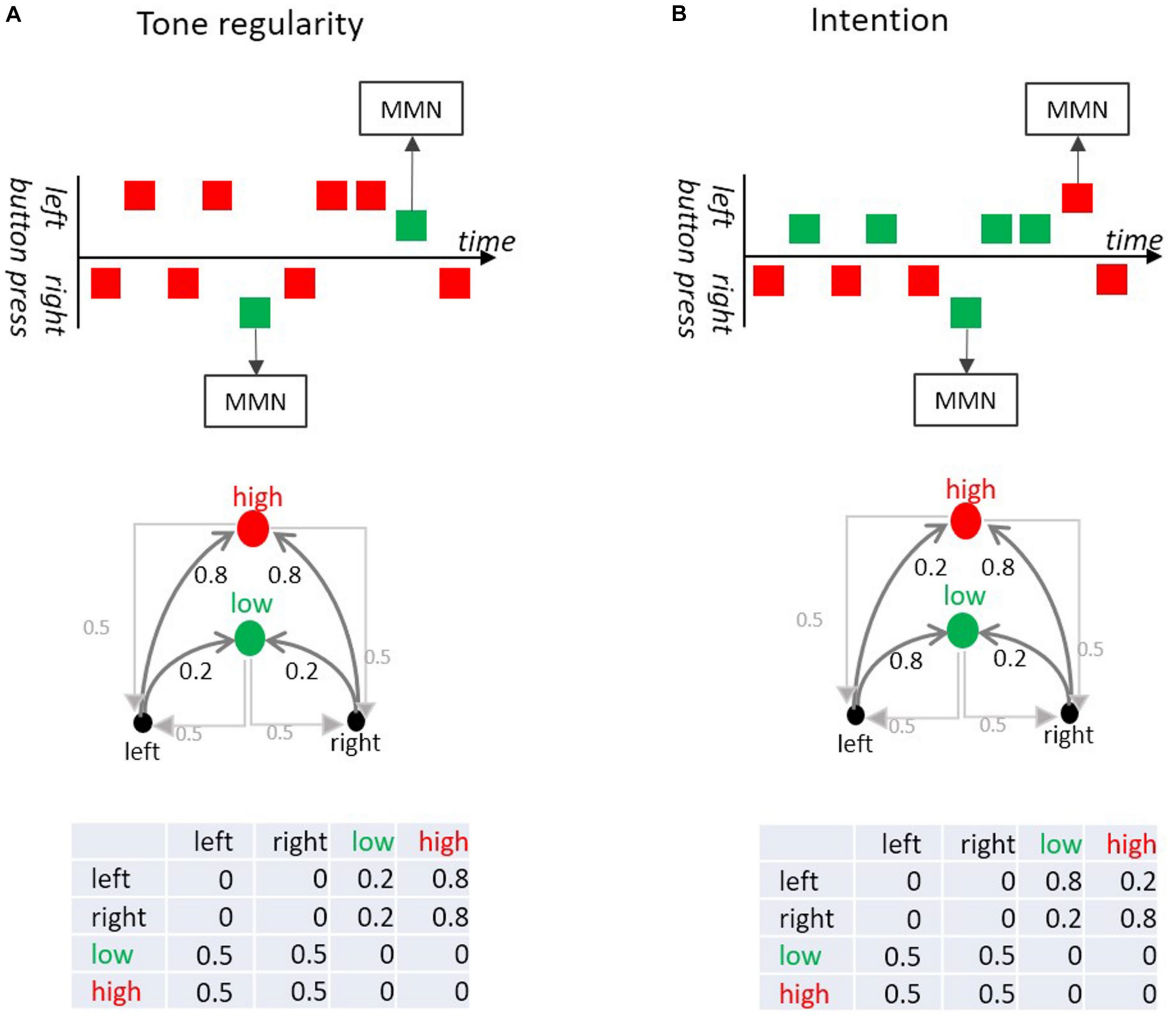
**(A)** In the Tone regularity condition, participants are instructed to press the left and right buttons equiprobably; each button press produces low (green) or a high (red) pitch tone. Most of the left and most of the right button presses produce a high pitch tone, but sometimes they produce a low pitch tone (deviant). The deviant tones (violating tone regularity) elicit MMN. **(B)** In the Intention condition, participants are instructed to generate the low tone with the left and the high tone with the right button equiprobably. In 20% of the trials a button press produces the “wrong” tone (i.e., high instead of low and vice versa). Although those deviants do not violate a tone regularity, they elicit MMN. This is because these wrong tones violate the intended action effect. The upper part of the figure shows excerpts of the sequences (adapted from Korka et al., 2019). The middle part shows the directed graphs of the Markov chains. The lower parts show the respective transition probability matrices.

Taking an experiment by Korka et al. (2019), we examine how the association between an action (button-press) and its sensorial consequence (tone) can be formalized by means of a transition matrix. Korka et al.’s participants randomly produced tones of different pitch via left and right button presses (Figure 7). In a “tone regularity” condition, both buttons produced a high-pitch tone with high probability and a low-pitch tone with low probability. In the “intention” condition the low-pitch tone was produced by a left-button-press and the high-pitch tone by a right button-press, such that both tones occurred with the same base rate. However, both buttons occasionally produced a tone associated with the opposite button (deviation from the button-tone association). Therefore, the tone sequence in the “tone regularity” condition consisted of an auditory standard and an auditory deviant at the level of pitch, while in the “intention” condition deviations occurred at the level of action-sound association (i.e., there were no pitch deviants in terms of base rate). Critically for our Markovian perspective, MMN was elicited both in response to low probability transitions between sounds (here, pitch deviants) and in response to low probability transitions between action and intended effect (here, button-tone deviants), that is even when the tone itself was not rare.

The finding that a sensorial prediction error signal like the MMN can be elicited in response to a sound conforming to a sensorial rule but violating an intended action-effect, is compatible with the predictive coding framework. That is because actions induce active (sensorial) inference to minimize prediction error (Friston and Stephan, 2007; Friston et al., 2010; Brown et al., 2013; Clark, 2013, 2015). In a recent study (Widmann and Schröger, 2022) further evidence was gathered that action-intention trumps base rate (or global sound probability). In this study participants were instructed to press buttons with an asymmetric frequency. That is, one button was to be pressed frequently and the other button only occasionally. Each button-press produced either a frequent or a rare tone, but the button-tone association was varied between experimental conditions. In a “predictable” condition the frequently pressed button produced the frequent tone and the rare button produced the rare sound. This association between button-press and tone was set-up such that participants produced tones corresponding to a self-generated classical oddball paradigm. As the frequently pressed button produced the standard and the infrequently pressed button the deviant, the occurrence of the pitch deviant was predictable for the participant. Although these deviants violated the auditory regularity defined by pitch, they did not elicit MMN. The most plausible conclusion is that the transition probability between the button-press and the sound (and not the transition probability between sounds) governed the MMN process. This finding also demonstrates clearly that action-intention can abolish prediction error at the level of MMN for a tone regularity deviant even when the action and the sound are rare. Interestingly, the N1 increase typically elicited in response to rare deviant tones compared to frequent standard tones was not affected (i.e., still present) by action-intention. This is further evidence that the Markovian perspective is compatible with the notion that transition probability between action and sounds is relevant to the brain. However, some prediction error signals (e.g., MMN) appear to be more sensitive to action-to-sound transition probability, while others (e.g., N1) are foremost subject to the base rate of the auditory stimuli. This shows that the brain considers more than just one statistic in modeling the world and that such information may exert differential effects along the processing hierarchy.

When we consider how existing computational models of MMN generation may explain the detection of sounds violating an intended action effect, we see a few differences. To our knowledge action-to-sound transitions have not yet been considered in the computational models of May and Tiitinen (2010), because their simulations were confined to auditory processes. The generic deviance detection principle by Chien et al. (2019), however, assumes that change detection relies on a common neural mechanism, namely reciprocal wiring, and is thus not an exclusively auditory phenomenon, but also applies to crossmodal situations. Lastly, in our view a generalized predictive coding theory including action (Friston et al., 2010; Clark, 2013) is the model most compatible with the experimental findings of action-sound coupling paradigms. This is, because the intended action effect can readily be regarded as a proxy for top-down prediction into the auditory processing level.

Apart from the salient action-to-sound transitions, the Markovian perspective reveals another aspect in action-sound-coupling paradigms when studying the lower left part of the transition matrices in Figure 7 that describes the study by Korka et al. (2019). Namely, there is not only a transition from button to sound, but also from sound to button (Aberbach-Goodman et al., 2022). In the example given here, the transition probability from any of the two sound types (high or low pitch) to any of the two types of button presses (left or right) is assumed to be 0.5. To avoid a bias in the button press types participants were instructed to randomly produce button presses but minding that, overall, left and right presses should be equiprobable. Yet, the sound-to-button transition is not entirely within the control of the experimenter, as participants may adjust their current action plan dependent on whether the action in the preceding trial yielded the intended action effect. That is, the outcome related to the button press in the preceding trial is potentially relevant to the preparation and/or execution of the button press in the current trial. A potential difference between match and mismatch of intended-action-effect and actually encountered sound in the preceding trial could manifest either or both in measures of motor behavior (e.g., speed, pressure), and brain activity (e.g., lateralized readiness potential, contingent negative variation). Indeed, such sequential effects have been studied to some extent (Pashler and Baylis, 1991; Notebaert and Soetens, 2003; Roeber et al., 2009; Varga et al., 2022). While it is beyond the scope of this paper to discuss sound-to-action transitions in detail, the brief example given here stresses the point that the Markovian narrative may trigger hypotheses and aid in the development of respective experimental designs.

## Conclusion, recommendations, limitations

The Markov model contains the accumulated knowledge about the succession of events. What happens next can be inferred from the respective transition matrix and the current state of the system. Similarly, for sequences of sensory events the brain can infer what is likely to happen next from detected regularities and the current event. Predictive regularity representations pertaining to probable and improbable transitions between events enable the differential processing of rule conforming and rule violating events, which, respectively, manifest as match and mismatch signals in the brain. This analogy might be somewhat simplistic, but it works out nicely (Figure 2). Yet, the statement that the processes underlying MMN behave like a Markov chain turned out to be neither completely true nor completely wrong. On the one hand, the hypothesis that MMN is only about transition probabilities is not completely true, because brain and behavioral responses measured for high transition probabilities but low base rates are rather different from those measured for high transition probabilities and high base rates (*cf.* Figure 4). On the other hand, the hypothesis that MMN is about transition probabilities is not completely wrong—for example, because a rare deviant tone with a pitch in between the pitches of the two frequent standard tones (high base rate) only elicits MMN if the standard tones are alternating (high transition probability), but not when they are presented randomly (low transition probability; Figure 6).

We introduced a taxonomy emphasizing transitions between events distinguishing three different types of MMN paradigms: classical oddball paradigms, between-sound regularity paradigms, and action-sound-coupling paradigms. Experimental data from all three types of paradigms are compatible with the Markovian perspective in many respects. However, when base rate of events is not controlled for (which in the MMN literature is largely the case), base rate can confound observed (ir)regularity effects. This shows that the brain’s attempts to model the world are not confined to transitions between successive events as a purely Markovian view might suggest, but that many different types of information facilitate the detection of invariances. Critically, our Markovian taxonomy provides a helpful tool to identify these sources of information—a transition matrix formalizes all potential transitions, thereby revealing potentially relevant contingencies in an experimental stimulation that may be overlooked otherwise. Moreover, findings both from classical oddball paradigms and from between-sound regularity paradigms can be accounted for by prediction-based accounts and primarily adaptation-based accounts, as both are able to simulate data from critical MMN experiments (e.g., omission paradigm, multi-standard paradigm). Moreover, although predictive coding accounts are already well conceptualized to model the processing of event sequences in action-sound-coupling paradigms, non-prediction-based accounts may possibly explain observations similarly well (Chien et al., 2019). While a Markovian view on MMN does not necessarily translate into a preference of one theoretical account of MMN generation over another, it can help make assumptions more explicit and thus, testable. For instance, whether base rate should matter, or whether frequency of transitions (base rate) should trump precision (transition probability).

Obviously, base probabilities of the sounds and transition probabilities between sounds are not completely independent from each other. That is, varying the event probability has an impact on the transition probabilities and vice versa. However, to a certain extent, one can be modulated without affecting the other, as can be seen in Figures 6A,B. In addition, sounds with high transition probabilities can be presented with rather low base probability (*cf.* Figure 4). This allows to design critical experiments, testing for the independent and combined contributions of base rate and transitions in (ir)regularity processing. This helps to distinguish different types of MMN mechanisms. To our knowledge, there are (almost) no studies considering all entries (and their combinations) in the transition probability matrix when designing and analyzing respective (ir)regularity experiments. This is a pity, as this could yield relevant information for MMN theory-considering Markov chains might reduce this shortcoming in experimental design.

Describing apparently different experimental frameworks in the common format of transition probability matrices, will also facilitate a more systematic comparison of the structure and the results of different studies. The Markovian view could therefore inspire new experimental ideas, for example, to investigate the brain’s processing of one specific transition rule that is embedded either in an overall more deterministic or in a more stochastic context. For instance, in an experiment with three different sounds one row in the transition probability matrix can be identical (rule of interest: B is followed in 80% by C and in 20% by A), but the other rows (transitions between other possible sounds) are deterministic (A is always followed by B and C is always followed by A) in one condition and stochastic (A is equiprobably followed by B and C; C is equiprobably followed by B and A) in the other condition.

Throughout this paper we pointed to potential limitations of a purely Markovian narrative (*cf.* section “Distinction from previous Markovian approaches to MMN (and related research)”). For example, a Markovian approach does not really care about the dynamics in the establishment and modification of predictive regularity representations as a function of the dynamics in the world and as a function of the constraints in the information processing system. Moreover, it is rather unspecific with regard to concrete neural networks achieving the brain’s fascinating ability to detect and use rules inherent to event sequences. Despite those limitations it seems promising to dig deeper in more specific subfields with this approach, for example, to consider the learning of the rules and the neural underpinnings. A broader look such as the present one, has two advantages: First, it may serve to sort the field according to more general principles. Second, it may foster exchange between rather different scientific disciplines (such as neurophysiology, computational modeling, and experimental psychology) on a joint topic such as the processing of sound sequences. In this paper, we shed light from the Markovian view on the MMN. However, the present approach can easily be generalized to other research fields where event sequences are comprised of sensory stimuli and/or behavioral responses such as implicit learning, sense of agency, theory of event coding, sensory-motor cycles, stimulus–response compatibility, associative learning, action-effect prediction, working memory, and others.

## Data availability statement

The original contributions presented in the study are included in the article/supplementary material, further inquiries can be directed to the corresponding author.

## Author contributions

ES developed the idea, wrote the initial draft, and compiled the figures. UR and NC commented the drafts, added text, and provided conceptual input. ES, UR, and NC read and approved the submitted version. All authors contributed to the article and approved the submitted version.

## Conflict of interest

The authors declare that the research was conducted in the absence of any commercial or financial relationships that could be construed as a potential conflict of interest.

## Publisher’s note

All claims expressed in this article are solely those of the authors and do not necessarily represent those of their affiliated organizations, or those of the publisher, the editors and the reviewers. Any product that may be evaluated in this article, or claim that may be made by its manufacturer, is not guaranteed or endorsed by the publisher.
